# Items and News

**Published:** 1852-12

**Authors:** 


*Items and News.*

Dr. Brown Sequard, during the months of September and October, gave highly interesting courses of lectures to classes of the most distinguished physicians in New York city. He is now engaged in the delivery of a similar course to the physicians of Boston.

Dr. F. W. Sargent has been appointed Surgeon to Will's Hospital, Philadelphia.

Dr. J. Lawrence Smith has been appointed Professor of Chemistry in the University of Virginia.

On a late trip of one of the Mississippi steamboats from New Orleans to St. Louis, the German emigrants on board opened a sack of castor beans, prepared and ate a huge luncheon of castor bean soup. The effect was, that in a short time the passengers in the cabin and offices of the boat were startled by the report, that the Cholera in its worst form had broken out on deck. The mystery was soon solved however, but the boat had to go into quarantine; because, as the Captain said, the Dutch "didn't know beans."

